# Empirical research in clinical supervision: a systematic review and suggestions for future studies

**DOI:** 10.1186/s40359-019-0327-7

**Published:** 2019-08-22

**Authors:** Franziska Kühne, Jana Maas, Sophia Wiesenthal, Florian Weck

**Affiliations:** Department of Psychology, Clinical Psychology and Psychotherapy, University of Potsdam, Karl-Liebknecht-Str. 24-25, 14476 Potsdam, Germany

**Keywords:** Supervision, Clinical supervision, Systematic review, Evidence-based psychotherapy

## Abstract

**Background:**

Although clinical supervision is considered to be a major component of the development and maintenance of psychotherapeutic competencies, and despite an increase in supervision research, the empirical evidence on the topic remains sparse.

**Methods:**

Because most previous reviews lack methodological rigor, we aimed to review the status and quality of the empirical literature on clinical supervision, and to provide suggestions for future research. MEDLINE, PsycInfo and the Web of Science Core Collection were searched and the review was conducted according to current guidelines. From the review results, we derived suggestions for future research on clinical supervision.

**Results:**

The systematic literature search identified 19 publications from 15 empirical studies. Taking into account the review results, the following suggestions for further research emerged: Supervision research would benefit from proper descriptions of how studies are conducted according to current guidelines, more methodologically rigorous empirical studies, the investigation of active supervision interventions, from taking diverse outcome domains into account, and from investigating supervision from a meta-theoretical perspective.

**Conclusions:**

In all, the systematic review supported the notion that supervision research often lags behind psychotherapy research in general. Still, the results offer detailed starting points for further supervision research.

**Trial registration:**

PROSPERO; CRD42017072606, registered on June 20, 2017.

## Background

Although in psychotherapy training and in profession-long learning, clinical supervision is regarded as one of the major components for change in psychotherapeutic competencies and expertise, its evidence base is still considered weak [1–3]. Clinical supervision is currently considered a distinct competency in need of professional training and systematic evaluation; however, theoretical developments and experience-driven practice still seem to diverge, and “significant gaps in the research base” are evident ([1], p. 88).

Definitions of supervision underline different aspects, whereas a lack of consensus seems to impede research [1]. Falender and Shafranske [4, 5] stress the development of testable psychotherapeutic competencies in the learners, i.e., their knowledge, skills and values/attitudes, through supervision; on the other hand, supervisors need to develop competence to deliver supervision. Milne and Watkins [6] describe clinical supervision as “the formal provision, by approved supervisors, of a relationship-based education and training that is work-focused and which manages, supports, develops and evaluates the work of colleague/s” (p. 4). In contrast, Bernard and Goodyear [7] emphasize supervision’s hierarchical approach, in as much as it is provided by more senior to more junior members of a profession. The goals of supervision may thus range between the poles of being normative (i.e., ensuring quality and case management), restorative (i.e., providing emotional and coping support) and formative (i.e., promoting therapeutic competence), and, thus, may ultimately lead to effective and safe psychotherapy [6]. Hence, it is pivotal for supervisors to reflect upon their own knowledge or skills gaps, and to engage in further qualification [8]. Clinical supervision may involve different therapeutic approaches and thus addresses therapists from varying mental health backgrounds [8], which is the stance taken in the current review.

Besides providing a definition of clinical supervision, it is relevant to delineate related terms. One is *feedback*, a supervision technique that “refers to the ‘timely and specific’ process of explicitly communicating information about performance” ([8], p. 28). Contrary to supervision, *coaching* strives to enhance well-being and performance in personal and work domains [9], and is therefore clearly distinct from supervision and psychotherapy with mental health patients provided by licensed therapists.

In the supervision literature, there is no paucity of narrative reviews, commentaries or concept papers. Previous reviews have revealed positive effects of supervision, for example on supervisee’s satisfaction, autonomy, awareness or self-efficacy [10–13]. Still, results on the impact of supervision on patient outcomes are still considered mixed [10]. Importantly, there is a knowledge gap regarding the active components of supervision, i.e., the effects of supervision or supervisor interventions on supervisees and their patients [10].

Past reviews, however, suffer from several limitations (for details, see [14]). First of all, strategies used for literature search and screening have not always been described or implemented rigorously, that is, implemented in accordance with the Preferred Reporting Items for Systematic Reviews and Meta-Analyses (PRISMA [15]) reporting guidelines (e.g. [10–12, 16–19]). Further, several reviews focus specifically on the positive effects of supervision [19] or specifically on learning disabilities [11], emphasize the authors’ point of view [20, 21], or concentrate on the supervisory relationship only [14]. While the majority of the above-mentioned reviews are narrative, Alfonsson and colleagues conducted a systematic review [14], pre-registered and published a review protocol [22] and implemented a thorough literature search and methodological appraisal. However, since they focused exclusively on cognitive behavioral supervision and on experimental designs, only five studies fit their inclusion criteria. Additionally, interrater agreement was only moderate during screening. Likewise, in our previous scoping review [23], we concentrated on cognitive behavioral supervision. Furthermore, like other supervision reviews [20, 21], it was published in German only, limiting its scope.

Thus, the current systematic review aimed to complement previous reviews by using a comprehensive methodology and concise reporting. First, we aimed to review the current status of supervision interventions (e.g., setting, session frequency, therapeutic background) and of the methodological quality of the empirical literature on clinical supervision. Second, we aimed to provide suggestions for future supervision research.

## Materials and methods

We conducted a systematic review by referring to the PRISMA reporting guidelines [15]. The review protocol was registered and published with the International Prospective Register of Systematic Reviews (PROSPERO; CRD42017072606).

### Inclusion and exclusion criteria

We included studies referring to clinical supervision as defined above by Milne and Watkins [6] above. Both, supervision conducted on its own or as part of a larger intervention (as in psychotherapy training) were included. Treatment studies in which supervision was conducted solely to foster treatment delivery were excluded because they mainly address study adherence and are still covered in other reviews [24, 25]. Furthermore, clinical supervision had to refer to psychotherapy, whereas supportive interventions accompanying other treatments (e.g., clinical management) were excluded. Thus, we included studies referring to mental health patients, and studies with patients with physical diseases were considered only if the reason for treatment was patients’ mental health. Studies with another population (e.g., simulated patients or pseudo-clients) were excluded. In order to focus the review in the heterogeneous field of clinical supervision, we limited it to adult patients. Studies on family therapy were included if they focused on adults. Studies with mixed adult and child/adolescent populations were included if the results were reported for the adult population separately. No prerequisites were predefined for supervisor qualification. Any empirical study published within a peer-reviewed process (i.e., without commentaries or reviews) and any outcome measures were included. As such, any supervision outcome (e.g., supervisees’ satisfaction or competence), including negative or unexpected outcomes (e.g., non-disclosure), were allowed. In line with Hill & Knox [10], we did not focus on studies exclusively examining the supervision process because firstly, it does not provide knowledge on the effectiveness of supervision, and secondly, relationship variables are already covered by other reviews [11]. Thus, the review focused on supervision interventions, and studies exclusively focusing on the effects of relationship variables or attitudes between the supervisee and supervisor (i.e., as independent variables) were excluded. However, relationship variables were considered if they were considered as dependent variables in the primary studies.

### Study search

**Fig. 1:**
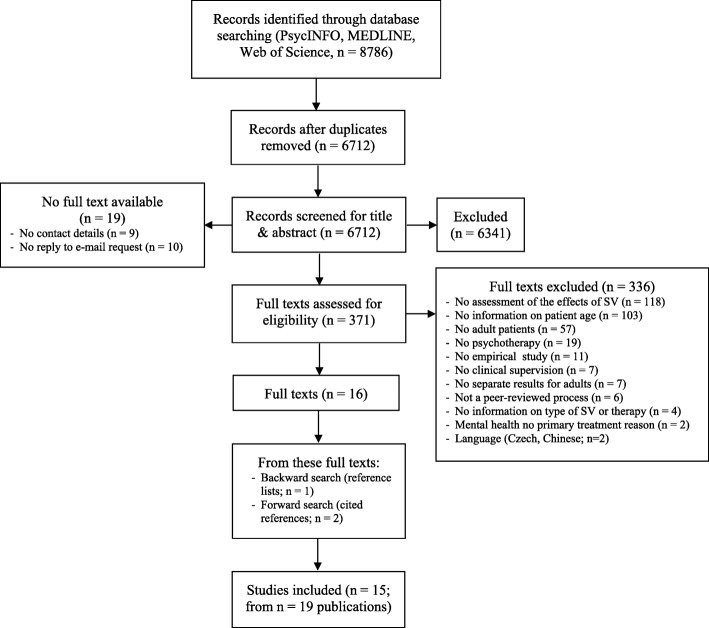
Flowchart on study selection. Adapted from Moher and colleagues (15); SV: supervision

The bibliographic database search was conducted during February and March 2017 in key electronic mental health databases (Fig. 1). To include the current evidence, we focused our search on studies published from 1996 onwards. There were no language restrictions. The following search strategy was used: supervis* AND (psychotherap* OR cognitive-behav* OR behav* therapy OR CBT OR psychodynamic OR psychoanaly* OR occupational therapy OR family therapy OR marital therapy) NOT (management OR employ* OR child* OR adolesc*). Then, we inspected the reference lists of the included studies (backward search) and conducted a cited reference search (forward search). We finished our search in July 2017.

### Screening and extraction

Referring to Perepletchikova, Treat and Kazdin [26], one reviewer (FK) introduced two Master’s psychology students (JM, SW) to the review methods, and the group discussed the review process in weekly one-hour sessions. First, titles and abstracts were screened for inclusion (JM, SW). The first 10% (*n* = 671) of all titles and abstracts were screened by both raters independently. Inter-rater agreement regarding title/abstract screening amounted to κ = .83 [CI = .73–.93], which is considered high [27].

Next, full texts of eligible and unclear studies were retrieved and then screened again independently by both raters (JM, SW). Disagreements were resolved through discussion or through the inclusion of a third reviewer (FK). If publications were not available through inter-library loans, a copy was requested from the corresponding author. For nine authors, contact details were not retrievable, and out of the 15 authors that were contacted, five replied. Inter-rater agreement concerning full text screenings for inclusion/exclusion was κ = .87 [CI = .77–.97].

For data extraction, we used a structured form that was piloted by three reviewers (FK, JM, SW) on five studies. It comprised information on supervision characteristics (e.g., setting, implementation and competence) and study characteristics (e.g., design, main outcome). Data were extracted independently by two raters, the results were then compared, and disagreements resolved again by mutual inspection of the original data.

### Methodological quality

Since we included various study designs, we could not refer to one common tool for the assessment of methodological quality. We therefore developed a comprehensive tool applicable to various study designs to allow for comparability between studies. For the development, we followed prominent recommendations [27–29]. The items were as follows: a) an appropriate design regarding the study question; b) the selection of participants; c) measurement of variables/data collection; d) control/consideration of confounding variables; and e) other sources of bias (such as allegiance bias or conflicts of interest). Every item was rated on whether low (1), medium (2) or high (3) threats to the methodological quality were supposed. The resulting sum score ranges from 5 to 15, with higher values indicating the possibility of greater threats to the methodological quality. The methodological quality was rated by two review authors independently (JM or SW and FK). Inter-rater reliability for the sum scores reached ICC _(1, 2)_ = .88 [CI = .70–.95], which is considered high [30]. Disagreements in ratings were again resolved through discussion within the review group.

Due to the heterogeneity of the study designs and outcomes, we will present the review results narratively and in clearly arranged evidence tables.

## Results

### Current status of supervision

#### Psychotherapies

Overall, 15 empirical studies allocated to 19 publications were included (Fig. 1). Information on the supervision characteristics is reported on the study level (Table 1). Most of the supervisees used cognitive-behavioral therapy (CBT) as the active intervention [35, 37, 39, 40, 43–45], in four studies, specific interventions such as Motivational Interviewing (MI [38, 42]), Dialectical Behavioral Therapy (DBT [41];) or Problem Solving Treatment (PST [32]) were used, and one study referred to psychodynamic therapy [31] (recommendation to “Conduct supervision from a meta-theoretical perspective”).

**Table 1:** Supervision characteristics (main studies reported in alphabetical order)

Publication	Therapy	Set-ting	Main mental health problem	SV manual or SVor training	Profession SVor	Therapy manual or SVee training	Profession SVee	Competence level SVee (%)				Supervision				
								Und	Grad	Post	PhD	Interven-tion	Frequency	Con-tact	For-mat	Tech-nology
Anderson (2012) [31]	PD	O	Depr, anx	–	Manual authors	Manual, instruction, video examples	Licensed PST	–	–	–	100	CD	Weekly / 1,5 yrs	F-t-f	Gr	Audio, video
Bambling (2006) [32]	PST	O	Major depr	Workshop, manual	Graduated in mental health, experienced	Workshop, manual	PS, PST, MHW, SW	–	20	75	5	CD	1 pre-PST + 7 weekly	F-t-f	Ind	–
Davidson (2017) [33]	Psychol-Therapy	O	Depr, anx, stress	–	PS, MHW	–	PS, MHW	–	–	–	–	FBO	–	F-t-f	Ind	–
Grossl (2014) [34]	Mixed	O	Mental disorders	–	PhD (PS, MFT)	–	Clinical PS, MFT, CS	–	68	32	–	FBO	16 weekly	F-t-f	Ind	–
Hiltunen (2013) [35]	CBT	O	Minor mental health problems	–	PST, experienced	Training	PS	–	100	–	–	–	3 weekly	F-t-f	Gr	Audio,video
Locke (2001) [36]	Mixed	O	–	–	Licensed	Training	–	–	–	–	–	CD, FBP	–	Live	Gr	Video, phone
Lu (2012) [37]	CBT	O	Comorbid PTSD	Workshop	PhD (PS)	Workshop	PS, CS, NU, SW	–	4	92	4	CD, FBP, RP, expert call	12-16x/weekly	F-t-f	Gr	Audio
Martino (2016) [38]	MI	O	Substance abuse	Workshop, manual, textbook	Certified, licensed CS	Workshop, manual, textbook	Substance abuse CS	9	14	68	1	FBP, coaching	On average 6.5x	F-t-f	Ind	Audio
Milne (2011) [39]	CBT	O	Depr, anx	Manual, training	Licensed PS	CBT training	PS	–	–	50	50	RP, FBP, others	37x / 11 months	F-t-f	Ind	Audio
Ng (2007) [40]	CBT	O	Medication-resistant psychosis	–	Certified PT, the CBT trainer	Manual, lectures	NU, SW	–	75	25	–	CD, RP	Weekly / 6 months	F-t-f	Gr	Video
Rizvi (2016) [41]	DBT	O	BPD	–	Licensed clinical PS / DBT expert / study author	Training, seminars	Clinical PS	–	–	100	–	FBP	5x weekly	BITE	Ind	PC, webcam
Smith (2012) [42]	MI	O, I	Substance abuse	Workshop	PhD (clinical PS)	Workshop	Substance abuse CS	28	31	40	–	CD, coaching	5x / 7 weeks	Live	Ind	Earpiece, phone
Weck (2016) [43]	CBT	O	Depr, anx	Technical instruction	Licensed clinical PS & SVors	CBT training	Clinical PS	–	–	100	–	CD, FBP	6x monthly	F-t-f, BITE	Ind	PC, webcam
Willutzki (2005) [44]	CBT	O	Affective disorders, anx	–	–	Training	PS	–	–	100	–	–	Every 4th session	F-t-f	Ind	–
Zarbock (2009) [45]	CBT	O	Affective & phobic disorders, others	–	Experienced	Training	PS	–	–	100	–	–	–	–	–	–

*SV* supervision, *SVsor* supervisor, *SVee* supervisee; − not applicable or no information, *PD* psychodynamic, *PST* problem-solving therapy, *CBT* cognitive behavior therapy, *DBT* dialectical behavior therapy, *MI* motivational interviewing, *Mixed* different approaches, *Psychol-Therapy* psychological therapy, not specified, *O* outpatient, *I* inpatient; *Depr* depression, *Anx* anxiety, *PTSD* post-traumatic stress disorder, *BPD* borderline personality disorder, *PST* psychotherapist, *PS* psychologist, *MHW* mental health worker, *SW* social worker, *MFT* marriage and family therapist, *CS* counselor, *PT* psychiatrist, *NU* nurse, *Und* undergraduate, no degree, student, *Grad* graduate, Bachelor degree, *Post* postgraduate, Master’s degree, *PhD* doctoral degree, *CD* case discussion, *FBO* feedback on patient outcome, *FBP* feedback on performance, *RP* role play, *coaching* provide model behavior, suggest statements, *F-t-f* face-to-face, *BITE* bug-in-the-eye, *Gr* group, *Ind* individual

#### Supervisions

Only a minority of studies described any form of supervision manual used or any prior training of supervisors [32, 37–39, 42, 43]. In most cases, supervisees were postgraduates or had a PhD degree. Regarding the frequency of supervision sessions, most studies reported weekly sessions [31, 32, 34, 35, 37, 41, 42], and the total number varied considerably from 3 [35] to 78 sessions [31]. Three studies did not describe the supervision frequency [33, 36, 45], and one singled out one supervision session only [44] (recommendation to “Describe how the study is conducted”).

#### Interventions

Whereas different forms of feedback or multiple-component supervision interventions were commonly studied, active interventions such as role play were seldom used [37, 39, 40]. Three studies did not describe the interventions used within supervision [35, 44, 45] (recommendation to “Investigate active supervision methods”). Four supervisions used a form of live intervention [36, 41–43], and the remainder conducted supervision face-to-face. All but five studies [32–34, 44, 45] investigated some form of technological support.

### Methodological quality

#### Design

The following sections describe the methodologies used in the studies, which is why all 19 publications are now referred to (Table 2). Five were randomized controlled trials (RCTs [32, 34, 38, 42, 43];), and one was a cluster-RCT [34]. In addition to cohort designs [31, 44], cross-sectional designs were common [35–37, 45, 48, 49]. Only in three publications was follow-up data collected [33, 38, 42]. Most studies covering satisfaction with supervision included one assessment time, usually post-intervention [34, 35, 37, 39, 48, 49].

**Table 2:** Study characteristics (main studies (bold type) and concomitant publications reported together)

Publication	Design	Intervention group (*n* patient)	Control group (*n* patient)	Ass	Q	Main outcomes	Negative effects
Anderson (2012) [31]	Cohort	Cohort year 2: Time-limited PD-SV Cohort year 3: Some early SV	Cohort year 1: No SV (84 all groups)	R	12	Sign. better adherence, therapeutic relationship and advanced PD techniques in PD-SV Small effects that do not seem sustainable	N/S
Anderson (2017) [46]	Cohort	Directive SV	Non-directive SV (40 both groups)	R	11	Sign. greater adherence of SVees if SVor used directive style Alternative explanations: SVor personality, didactic methods, individual differences of SVees	–
Bambling (2006) [32]	RCT	Alliance process-focused SV (34) Alliance skill-focused SV (31)	No SV (38)	Q, R	9	Sign. increased therapeutic alliance and decreased depression in all groups, group differences after session 1 Pat. in SV groups sign. More satisfied and less dropout than in control group	–
Davidson (2017) [33]	Cluster RCT	FB to SVee and SVor on Pat outcome, alerts as to worsening (16)	FB to SVee on Pat outcome, no alert (25)	Q	13	Pat. in control group sign. Less distressed (post, FU), also in therapists’ ratings, but with more sessions Large pat. and therapist drop-out	risk for self-harm evaluated
Grossl (2014) [34]	RCT	FB to SVee and SVor on Pat outcome	SAU (138 both groups)	Q	12	N.s. differences between groups SVees in intervention group sign. More satisfied with SV	–
Hiltunen (2013) [35]	CS	CBT-SAU (35)	–	Q	13	Perceived satisfaction with SV	–
Locke (2001) [36]	CS	Live-SV (108)	–	Q	13	Pat. felt comfortable with Live-SV Perceived helpfulness and low intrusiveness of Live-SV predicted therapy satisfaction	–
Lu (2012) [37]	CS	CBT-SV with fidelity FB (26)	–	Q, R	14	SV and E-mail FB perceived as helpful, pat. Symptoms sign. Decreased 91% of SVees achieved certification with first training case	–
Martino (2016) [38]	RCT	SV on MI (227)	SAU (223)	R, I, T	10	Sign. greater increase in SVee competency in intervention group (post, FU) N.s. differences in pat. Retention and substance abuse, MI-SV more cost-intensive	27 adverse events, unrelated
Milne (2011) [39]	*N* = 1 (ABA)	B: Evidence-based clinical SV (3)	A: CBT-SV	Q, R, I, O	14	Intervention perceived as better, experiential learning and high acceptance in both groups	Anxious, rushed, taxing
Milne (2013) [47]	S/A	S/A	S/A	R	15	Apparent SVor fidelity and perceived experiential learning in SVees	–
Ng (2007) [40]	Pre- post	SV to CBT for psychosis (10)	–	R, CF	15	More acceptable case formulations and sign. Better therapeutic competences after SV	–
Rizvi (2016) [41]	N = 1 (ABA)	B: BITE-SV (1)	A: SAU	Q, R	11	Pat/SVee perceived BITE as acceptable, SVee perceived increase in DBT confidence, adequate adherence	–
Smith (2012) [42]	RCT	Live phone-SV on MI with standardized Pat	Audiotape-based phone-SV on MI with standard. Pat; No SV	R	10	Intervention with sign. Greater global MI integrity and skill than Audiotape-based SV than No SV Audiotape-based SV sign. Better in increasing complex reflections	N/S
Weck (2016) [43]	RCT	BITE-SV (19)	Delayed video-based SAU (23)	Q, R	11	Sign. better therapeutic alliance and competence in intervention group N.s. differences when controlling for baseline scores and for pat. Outcomes	–
Jakob (2013) [48]	CS	BITE subgroup (10)	–	Q	13	High acceptance, perceived helpfulness and usefulness by Pat, SVee, SVors	Split attention
Jakob (2015) [49]	CS	BITE subgroup (8)	–	I	10	Positive perception of an added value by BITE e.g., on therapeutic competence For SVees, organizational efforts and anxiety at the beginning	Stress
Willutzki (2005) [44]	Cohort	Additionally requested CBT-SAU	Regular CBT-SAU (104 in total cohort)	Q	14	Perceived problematic therapeutic alliance before additionally requested SV (Pat, SVee) Small effects on improved therapeutic alliance after SV, high satisfaction with SV	–
Zarbock (2009) [45]	CS	SAU: Multimodal BT (90)	–	Q	13	Supervisory relationship as best predictor of overall SV satisfaction Low correlation between SVor and SVee ratings of SV	–

*Ass* assessment methods, *SV* supervision, *SVsor* supervisor, *SVee* supervisee, *SAU* supervision as usual, *Pat* patient, − not applicable or no information, *S/A* see above, *N/S* indicated but not specified, *PD* psychodynamic, *C/BT* cognitive / behavior therapy, *DBT* dialectical behavior therapy, *MI* motivational interviewing, *RCT* randomized-controlled trial, *CS* cross-sectional study, *N* = 1 N of 1 trial, ABA withdrawal); *Rat* rating, *Ques* questionnaire, *Int* interview, *Obs* observation, *T* test, *CF* case formulation, *FB* feedback, *MI* motivational interviewing, *BITE* bug-in-the-eye, *FU* follow-up, *N.s./sign*. non/significant, *Q* methodological quality, 5 (lowest) to 15 (highest possible threat

#### Methodological quality

**Fig. 2:**
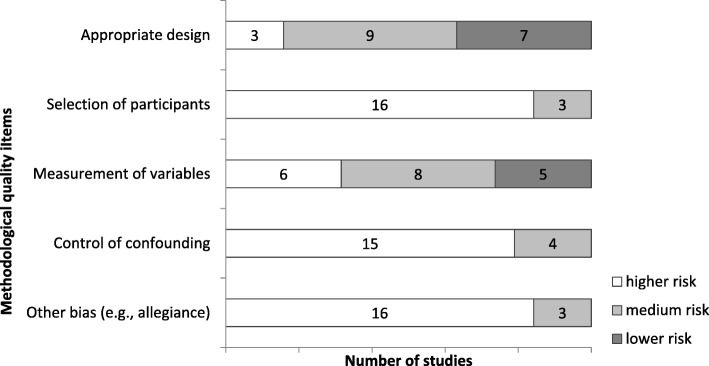
Methodological quality of the included studies. Lower *risk* … lower *possible threats to methodological quality*, sum score of 9–11 (range 5–15); medium risk … 12–13; higher risk … 14–15; e.g., 16 studies with higher risk of threats regarding selection of participant issues

The assessments of the methodological quality are presented in Table 2. The total methodological quality score was between 9 and 11 in six publications [32, 38, 41–43, 46, 49], between 12 and 13 in eight publications (score of 12–12 [31, 33–36, 45, 49];), and between 14 and 15 in five of the 19 publications [37, 39, 40, 44, 47], with a lower score indicating a lower risk of a threat to the methodological quality. On an item level, most problems referred to the selection of participants, the control of confounders, and other bias such as allegiance bias (Fig. 2; recommendation to “Conduct methodologically stringent empirical studies”).

### Effects of clinical supervision

**Fig. 3:**
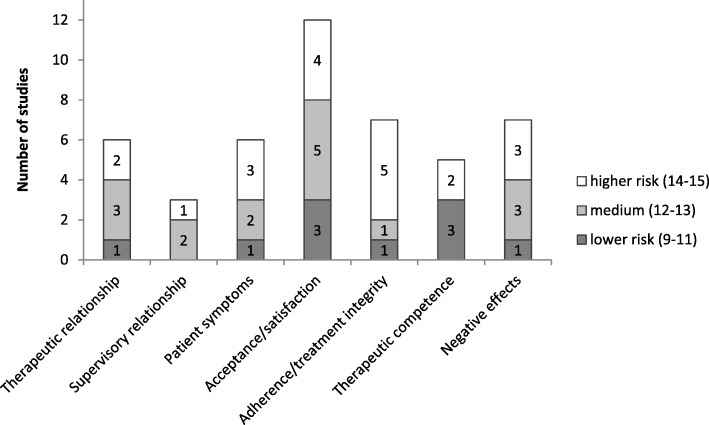
Supervision outcomes and methodological quality of the respective studies. In relation to the methodological quality; e.g., 2 studies with medium and 1 study with higher risk of possible threats to methodological quality investigated the supervisory relationship

The most consistent result refers to the high acceptance, satisfaction and the perceived helpfulness of supervision by supervisees [34–37, 39, 41, 44, 48, 49]. Further, the therapeutic relationship [31, 32, 43–45], and therapeutic competence seem to benefit from supervision [37, 38, 40, 42, 43]. On the other hand, non-significant findings [34, 38], small effects [31, 44, 45] and relevant alternative explanations [32, 33, 43, 46] hamper proper conclusions (see Fig. 3).

Whereas most publications did not describe negative or unexpected effects of supervision, two mentioned them without further specification [31, 42], two referred to unwanted effects as being unrelated to the outcome [33, 38], and three described limits to therapists’ cognitive capacity and perceived anxiety or stress during supervision [39, 48, 49] (recommendation to “Investigate diverse positive and negative supervision outcomes aside from acceptance”).

## Discussion

The aim of the present study was to systematically review the status and quality of the current empirical literature on clinical supervision and, based on the review findings, to draw conclusions for future studies. The current review identified 19 publications referring to 15 empirical studies on the status of clinical supervision. Despite using wide inclusion criteria, it is remarkable that only such a small number of studies could be included. In contrast to former reviews, our study was conducted systematically according to current guidelines, using a reproducible methodology and concise reporting. Compared to previous reviews, it was not limited to psychotherapeutic approaches or study designs.

Regarding the psychotherapeutic approaches of the supervisees, most interventions had a CBT background, which still documents a research gap in studies on clinical supervision between CBT and other therapeutic approaches.

Aside from psychotherapy approaches, the meta-theoretical perspective of competency-based supervision, as proposed by the American Psychological Association [8], provides a more integrative and broader view. Their supervision guidelines involve seven key domains central to good-quality supervision, from supervisor competencies to diversity or ethical issues. Importantly, they describe supervision to be science-informed, which again underlines the importance of supervisors and supervisees to keep their evidence-based knowledge and skills up-to-date during profession-long learning.

Considering the conduction of supervision, face-to-face supervision was prevalent, but technological support was common as well, at least in published empirical studies. A variety of interventions was used, including less active ones such as case discussions and coaching, as well as more active ones such as feedback on patient outcomes or supervisee performance. It is clearly positive that active interventions (such as coaching and feedback) were implemented and evaluated because they have proven useful in active learning and therapist training [50]. Nevertheless, even more active methods, such as exercise or role play, were an exception [23]. Furthermore, it remains unclear which interventions are helpful in profession-long learning and maintenance of expertise [21, 23]. We found that central supervision characteristics, such as the training of supervisors or the manual used for supervision, were not described consistently. Although a detailed description of how studies were conducted seems intuitive, it is surprising that reporting guidelines are not referred to consistently.

Concerning design characteristics, most studies were uncontrolled or used small samples. Further constraints were associated with the lack of follow-up data and major inconsistencies in the evaluation of negative effects. Although external observers, which were only sometimes independent, were used, almost half of the studies relied exclusively on self-reported questionnaires. Another problem was that the heterogeneity in the designs and instruments hampered the quantitative summary of results. Methodological quality has been criticized in supervision research for years (e.g. [16, 17],), and inconclusive findings or relevant alternative explanations additionally impeded firm conclusions on supervision effects. Regarding the effects of clinical supervision, the review documents that supervision research clearly lags behind psychotherapy research in general; that is, we still have limited evidence on supervision effects, especially those regarding patient benefits [10], and we continue to search for active supervision ingredients [51].

Acceptance and satisfaction are crucial prerequisites for supervision effects, and they were the variables most frequently investigated. Although positive results in these domains may be considered stable [13], satisfaction may not be confused with effectiveness. Taken from health care-related conceptualizations [52], subjective satisfaction may depend on a number of variables, such as mutual expectations, communication, the supervisory relationship, the access to supervision or financial strains. In this sense, satisfaction is distinct from learning and competence development. Other important outcomes of supervision, such as the therapeutic relationship and competencies, treatment integrity, patient symptoms or unwanted effects, clearly need further investigation [10, 21]. Other ideas include considering not only the supervisory relationship but also supervisory expectations as important process variables across psychotherapeutic approaches [13].

### Limitations

We constructed a short tool for rating methodological quality, which enabled comparisons between the diverse designs of the studies included. Although inter-rater reliability was high, it lacks comparability with other reviews. Due to a stricter operationalization of the inclusion criteria, six studies were included in our previous scoping review [23], and three were included in another current review [14] that were not part of the current systematic review. More specifically, one study was not located via our search strategy, and the other publications did not describe explicitly if the patients were adults. As the excluded publications were mainly referring to CBT supervision, it generally reflects the stronger evidence-base of CBT that has its roots in basic research. Since the review aimed to illustrate the status and quality of supervision research, we did not restrict it to specific designs, but mapped the status quo. This necessarily increased heterogeneity, and especially regarding supervision effects, it limited the possibility to draw clear-cut conclusions or to combine the results statistically. Differences in the results of reviews may result not only from methodological aspects but also from diversity in the primary studies, which may be addressed only by better supervision research [14].

## Conclusions

The review provides a variety of starting points for future research. The recommendations derived mainly refer to the replicability of research (i.e., to conduct methodologically stringent empirical studies, and to include positive and negative supervision outcomes). Taking a competency-based view, the following are examples of significant foci of both future practice and supervision research [23, 53, 54]:

- Define, review and continuously develop supervisor competencies.
- Include active methods, live feedback and video-based supervision.
- Enhance the deliberate commitment to ethical standards to protect patients.
- Positively value and include scientific knowledge and progress.
- Foster profession-long learning of supervisees and supervisors.

Logistics may be an important issue in supervision research. Therefore, if large-scale quantitative studies are difficult to conduct or fund, methodologically sound pragmatic trials [3] and experimental studies may be feasible alternatives. Most of the results still speak to the lack of scientific rigor in supervision research. Thus, we consider competency-based supervision and research investigating the essential components of supervision as the major goals for future supervision research and practice.

## Data Availability

All data generated or analyzed during this study are included in the published article.

## Abbreviations

CBT: Cognitive-behavioral therapy
DBT: Dialectical behavioral therapy
MI: Motivational interviewing
PRISMA: Preferred reporting items for systematic reviews and meta-analyses
PROSPERO: International prospective register of systematic reviews
PST: Problem solving treatment
RCT: Randomized controlled trial
SV: Supervision
